# Diagnostic Nerve Block to Guide Botulinum Neurotoxin Type A Injection for Clonus in Spastic Equinovarus Foot: A Retrospective Study

**DOI:** 10.3390/toxins16120503

**Published:** 2024-11-21

**Authors:** Mirko Filippetti, Stefano Tamburin, Ilaria Di Maria, Cecilia Angeli, Rita Di Censo, Elisa Mantovani, Nicola Smania, Alessandro Picelli

**Affiliations:** 1 Department of Neurosciences, Biomedicine and Movement Sciences, University of Verona, 37100 Verona, Italy; mirko.filippetti@univr.it (M.F.); ilaria.dimaria_02@studenti.univr.it (I.D.M.); cecilia.angeli@studenti.univr.it (C.A.); rita.dicenso@univr.it (R.D.C.); elisa.mantovani@univr.it (E.M.); nicola.smania@univr.it (N.S.); alessandro.picelli@univr.it (A.P.); 2 Canadian Advances in Neuro-Orthopedics for Spasticity Consortium (CANOSC), Kingston, ON K7K 1Z6, Canada; 3 Neurorehabilitation Unit, Department of Neurosciences, University Hospital of Verona, 37100 Verona, Italy

**Keywords:** botulinum toxins, clinical decision making, muscle spasticity, nerve block, ultrasonography

## Abstract

Clonus is characterized by involuntary, rhythmic, oscillatory muscle contractions, typically triggered by rapid muscle stretching and is frequently associated with spastic equinovarus foot (SEVF), where it may increase risk of falls and cause discomfort, pain, and sleep disorders. We hypothesize that selective diagnostic nerve block (DNB) of the tibial nerve motor branches can help identify which muscle is primarily responsible for clonus in patients with SEVF and provide useful information for botulinum neurotoxin type A (BoNT-A) treatment. This retrospective study explored which calf muscles contributed to clonus in 91 patients with SEFV after stroke (n = 31), multiple sclerosis (n = 21), and cerebral palsy (n = 39), using selective DNB. We found that SEVF-associated clonus was most commonly driven by the soleus muscle, followed by the gastrocnemius lateralis and medialis, tibialis posterior, and flexor digitorum longus, and that frequency differed according to SEVF etiology. Our data suggest that identifying the muscles involved in SEVF-associated clonus may aid clinicians in personalizing BoNT-A treatment to single patients. Also, the findings of this study suggest that applying a ‘stroke model’ to treating spasticity secondary to other etiologies may not always be appropriate.

## 1. Introduction

Equinovarus foot is one of the most common lower limb spasticity patterns and is often part of the typical posture observed in these individuals, i.e., equinovarus foot with flexed toes [1,2,3,4]. Spastic equinovarus foot (SEVF) can be attributed to four primary factors: spasticity, affecting the soleus, gastrocnemius, tibialis posterior, flexor digitorum, and flexor hallucis longus muscles [3]; muscle shortening; muscle weakness; and imbalance [5].

Spasticity often occurs alongside clonus, both of which are positive symptoms of the upper motor neuron syndrome [6]. Clonus is characterized by involuntary, rhythmic, oscillatory muscle contractions, typically triggered by rapid muscle stretching [7,8,9]. Ankle involvement is the most frequently observed presentation of clonus in spasticity. The underlying physiopathological mechanism of clonus remains a subject of debate. Although clonus generally results from alternating contractions of opposing muscle groups, not all studies have convincingly demonstrated antagonist muscle activity. One theory suggests that clonus arises from an exaggerated stretch reflex, particularly in the triceps surae, as supported by evidence of absent antagonist electromyography (EMG) activity [10,11]. Conversely, another hypothesis proposes that a central pattern generator within the spinal cord may be involved, as indicated by antagonist EMG muscle activity (e.g., in the tibialis anterior). Additional evidence points to a significant contribution from peripheral mechanisms [12], and this view is supported by the effectiveness of cold therapy in reducing clonus, whereas central alpha-2 antagonists (e.g., tizanidine) have shown limited efficacy [9].

Clonus may impair active function, including maintaining an upright posture and walking safely with a consequent increased risk of falls, and may cause discomfort, pain, and sleep disorders. Together, these factors reduce overall quality of life and significantly contribute to disability [13].

Given the frequent association between clonus and the SEVF pattern of spasticity [14], identifying the contribution of each calf muscle to clonus could significantly aid clinicians in optimizing focal treatment with botulinum neurotoxin type A (BoNT-A), which is a first-line treatment for focal spastic muscle overactivity [3,15,16,17,18]. An accurate administration of BoNT-A—ensuring that the correct dose is injected into specific muscles involved in different spasticity patterns—is essential for achieving optimal outcomes in spasticity [19]. The diagnostic nerve block (DNB) technique has proven valuable in managing spasticity by distinguishing between muscle overactivity and contracture, by providing a temporary clinical and functional change that can help predict the effect of BoNT-A injection, neurolysis, or selective neurectomy. DNB also helps refine therapeutic goals per patient needs [20,21,22,23].

We hypothesized that selective DNB of the tibial nerve motor branches can help identify which muscle is primarily responsible for the clonus in patients with SEVF. This retrospective study had two aims. The primary aim was to explore the frequency of calf muscles contributing to clonus in patients with SEFV spasticity after stroke, multiple sclerosis (MS), and cerebral palsy (CP), using selective DNB techniques. The secondary aim was to provide information on the BoNT-A dose to control clonus in different muscles in SEVF. These pieces of information may help optimize decision making for BoNT-A treatment.

## 2. Results

Based on the inclusion and exclusion criteria, 91 patients were deemed eligible for this study from the analyzed medical records (January 2020–August 2024). Both lower limbs were often involved in patients with MS (n = 21) and CP (n = 39), and a total of 116 lower limbs were assessed. Table 1 summarizes the key demographic and clinical characteristics of the enrolled patients. Patients with CP were younger and with a longer spasticity duration, but a subset presented with spasticity for the first time in adulthood. There was an overall predominance of male patients across all groups, particularly in the stroke group.

Per the inclusion criteria, all patients had a pre-DNB Tardieu scale score median of 3 or higher, with the knee extended and flexed. Following the DNB procedure, all patients showed a complete resolution of clonus, with a post-DNB median of 2 in both conditions (Table 2).

Among the 116 lower limbs with SEVF and clonus, 165 muscles were identified as contributing to clonus. Frequency analysis showed that clonus was most commonly driven by the soleus muscle, followed by the gastrocnemius lateralis (GL) and gastrocnemius medialis (GM) muscles. The tibialis posterior (TP) muscle was identified as contributing to clonus slightly less frequently, while the flexor digitorum longus (FDL) was the least frequently involved (Table 3).

**Figure 1:**
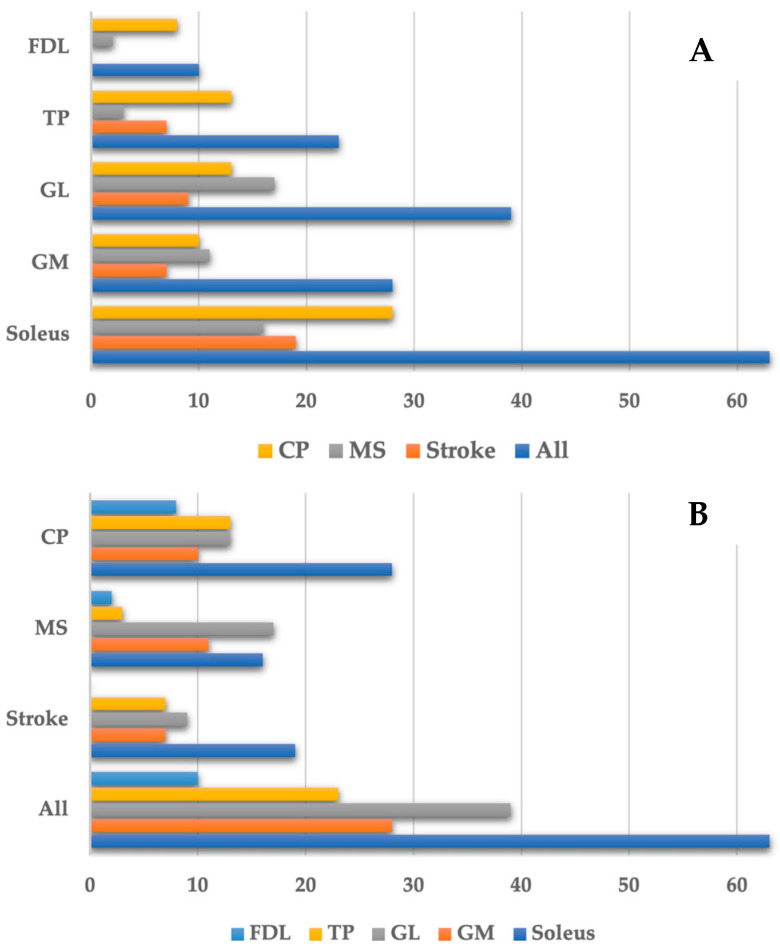
Frequency of muscles causing clonus grouped by muscle (**A**) and etiology of spasticity (**B**). CP: cerebral palsy; MS: multiple sclerosis; FDL: flexor digitorum longus; GM: gastrocnemius medialis; GL: gastrocnemius lateralis; TP: tibialis posterior.

When stratified according to the etiology of spasticity, clonus was predominantly caused by the soleus, followed by the GL, then the TP and GM after stroke, with no contribution of the FDL. In patients with MS, the GL and soleus showed similar frequencies, with the GL being slightly more frequently involved, and GM overactivity was the third most common cause of clonus. In patients with CP, the soleus was the primary contributor, followed by equal involvement of the GL and TP, with the GM and FDL contributing less frequently (Table 3, Figure 1).

Interestingly, among the 91 patients enrolled, one patient with hemorrhagic stroke exhibited an SEVF pattern accompanied by an atypical presentation of clonus. Initially, a DNB was performed on the tibialis nerve motor branch for the TP muscle, resulting in a partial response, with the clonus transitioning from sustained to un-sustained (i.e., from Tardieu grade 4 to 3). However, after the DNB for the TP muscle, the clonus did not follow the typical plantar–dorsiflexion pattern, instead manifesting in a mediolateral direction, suggesting the involvement of the peroneal muscles. The clonus resolved after performing a DNB of the peroneal nerve (i.e., from Tardieu grade 3 to 2).

Table 4 reports the mean BoNT-A dose administered for each muscle. Soleus and TP were the muscles injected with the higher and lower BoNT-A dose, respectively. One month after the BoNT-A treatment, the Tardieu grade demonstrated a median of 2 at knee extended and flexed (Table 4).

## 3. Discussion

The SEVF is the most common disabling pattern of lower limb spasticity [3]. Ankle clonus is the most frequent form of clonus and is frequently associated with SEVF, in both knee-flexed and knee-extended positions [14]. SEVF significantly affects gait parameters, negatively impacting all step phases [6], increasing pain and the risk of falls during walking. The presence of clonus further exacerbates disability. Deltombe and colleagues have already established the crucial role of DNB in managing SEVF in the Mont-Godinne guidance pathway [5]. Additionally, recent findings have questioned the validity of the Silfverskiöld test in identifying the specific muscle that, within the triceps surae, is responsible for the SEVF pattern in knee-flexed and extended conditions [25]. As a result, DNB has gained growing importance in helping clinicians identify which muscle sustains clonus in the SEVF pattern. In this regard, a pivotal study by Decq and collaborators reported that, in a sample of patients with SEVF and clonus, soleus spasticity accounted for 75% of clonus cases, with 12.5% due to the gastrocnemius (without specifying whether the GM or GL were involved), and 12.5% to both the soleus and gastrocnemius [14]. Our findings largely align with these data. However, the study by Decq and colleagues [14] involved only 16 patients with spasticity due to various etiologies (stroke, n = 5; MS, n = 1; spinal cord injury, n = 4; cervical myelopathy, n = 2; other causes, n = 4) [14], limiting the possibility to perform stratified analyses according to the underlying etiology. Although the frequency of clonus was not described, Filippetti and colleagues [26] reported a similar ranking of the triceps surae muscles sustaining the SEVF pattern in 48 patients accounting for a total of 64 different DNBs with various etiologies (stroke, n = 22; MS, n = 9; brain injury, n = 7; spinal cord injury, n = 6; other causes, n = 4), with the following distribution of muscles involved: soleus, 53%; GL, 11%; GM, 13.5%; and TP, 23.5%.

Our data further confirm that the soleus muscle, either alone or in combination with other calf muscles, is the most common muscle sustaining clonus (38.2%), followed by the GL (23.6%) and GM (17.0%) muscles. This pattern is generally consistent across the three etiologies of spasticity examined in this study. The rank order of muscles involved in clonus was soleus, GL, GM, and TP in patients with stroke; soleus, GL, and TP equally, GM, and FDL in patients with CP; and GL, soleus, GM, TP, and FDL in patients with MS. Based on this evidence, the roles of other plantar flexor muscles, i.e., the GL, GM, and TP in sustaining clonus, are not secondary. Notably, among patients with MS, clonus in the GL slightly exceeded that in the soleus (34.7% vs. 32.7%). Additionally, in patients with CP, clonus due to the TP was equal in frequency to that of the GL (18.1%). These findings slightly contrast with the recommendations of Deltombe and collaborators, who stated that soleus spasticity is typically clonic and responsible for triceps clonus, while gastrocnemius spasticity tends to be more tonic and prone to spastic co-contraction [5]. The same guidelines suggest that, if DNB of the soleus provides incomplete relief, the next DNB should target the TP, followed by the GM and GL [5]. Our data suggest a partial revision of this model, prioritizing the GL in patients with stroke and CP, and the GL and GM in patients with MS, over the TP in cases of clonus associated with spasticity.

In the context of personalized treatment, identifying the muscles most frequently responsible for clonus in the SEVF pattern can help clinicians prioritize DNB or make BoNT-A injections more effective. Inappropriate muscle selection is not only a missed opportunity but may also worsen the patient’s disability, even if the effect is temporary.

The findings of this study further reinforce the notion that insights derived from the ‘stroke model’ of spasticity management cannot be universally applied to all etiologies leading to SEVF. This concept has been previously noted, particularly regarding SEVF, where Picelli and colleagues have documented differences in clinical features between stroke and MS patients [27]. Moreover, Baricich and collaborators [17] have emphasized the importance of tailoring BoNT-A treatment to the underlying cause of spasticity.

Although less common, the failure of BoNT-A treatment to resolve clonus should not be attributed solely to issues with injection technique or dose optimization. It is also essential to consider that clonus might be sustained by less frequently involved muscles, such as the TP and FDL. Clonus originating from atypical muscles should also be considered, although this is rare. In one patient included in this study, the peroneal muscles were found to contribute to the clonus. This is an uncommon occurrence, with only one other reported case in the literature, where clonus was driven by the peroneal muscles [28].

Based on the Tardieu scale, a beneficial effect in controlling clonus was observed in approximately 50% of patients, both with their knee flexed and extended, with a median score of 2. The lack of a 100% response among patients could be attributable to the optimal BoNT-A dose typically achieved over 2–3 injection cycles, which allow for adjusting the dose according to the clinical response. To our knowledge, there are no data on the mean dose administered or the efficacy of BoNT-A injection on clonus when guided by clinical evaluation. Thus, we cannot conclude if DNB may result in a more tailored BoNT-A dose and better control of clonus than clinical evaluation, and this should be the topic of future studies.

This study has several limitations. They include the retrospective design, the inability to infer the average optimal dose of BoNT-A required to control clonus in specific muscles due to a lack of dose data apart from those of the first injection after DNB, and the absence of a correlation between clonus and qualitative muscle characteristics, e.g., fibro-adipose degeneration according to the modified Heckmatt scale [29].

Although it is known that specific muscles (e.g., the soleus and GL) are more involved in the generation of clonus compared to other plantar-flexor muscles, the reason for this remains unclear. Furthermore, differences in clonus generation can be observed when investigating this phenomenon in the context of specific pathologies, with the underlying mechanisms still poorly understood. The different functional roles of the muscles (e.g., tonic in the soleus and phasic in the gastrocnemius muscles), as well as the varying degrees of involvement with the spastic myopathy and, consequently, the degree of the fibro-adipose degeneration of the muscle, may play a role in the genesis of clonus. However, these remain potential hypotheses for future studies rather than established evidence.

## 4. Conclusions

Identifying the muscles most frequently involved in sustaining clonus within common spasticity patterns, such as SEVF, can help clinicians determine which DNB to perform first and optimize BoNT-A treatment by enabling a more targeted therapeutic approach. Inaccurate treatment can not only represent a missed opportunity but also worsen function and increase disability. Furthermore, the findings of this study suggest that applying a ‘stroke model’ to the management of spasticity with clonus secondary to other etiologies may not always be appropriate.

## 5. Materials and Methods

### 5.1. Study Design

This retrospective study involved patients who underwent DNB assessment for SEVF. This study reviewed patients’ medical records referred for the first time to our clinical unit between January 2020 and August 2024.

The inclusion criteria for the study were as follows: (a) age 18 years or older, (b) diagnosis of SEVF secondary to stroke, cerebral palsy, or multiple sclerosis, (c) Tardieu grade 3 or 4, (d) modified Ashworth Scale (MAS) score < 4, and (e) completion of DNB assessment for SEVF. Patients were excluded if complete data from their clinical records could not be obtained.

**Figure 2:**
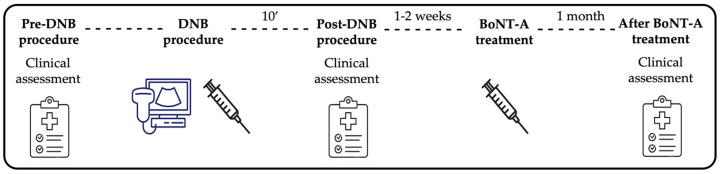
Timeline of the study.

Patients underwent (a) clinical assessment before and (b) after the DNB procedure; (c) BoNT-A treatment of the calf muscles, based on DNB and 1–2 weeks later; and (d) clinical assessment one month after BoNT-A injection (Figure 2). Baseline data (i.e., before performing DNB) included gender, age, the underlying cause of spasticity, and clinical features of SEVF using the Tardieu grade and MAS for both knee-extended and knee-flexed positions. Data collected approximately 10′ after each DNB procedure included the specific motor nerve branches targeted, the dose of lidocaine, and the Tardieu grade for both knee-extended and knee-flexed positions. The Tardieu grade was also assessed after BoNT-A treatment.

Two experienced clinicians in spasticity management conducted both clinical assessments and DNB procedures.

### 5.2. Clinical Assessment

Clinical evaluations were conducted with the patient lying supine on a bed, in accordance with standard clinical practice. The Tardieu grade was recorded at knee-extended and knee-flexed positions before and after each selective DNB procedure, and after BoNT-A injection. MAS scores were recorded before DNB according to the inclusion criteria. The Tardieu grade was used to evaluate the presence and the grade of clonus at the spastic calf muscle according to the spasticity grade, which measures the gain in muscle reaction to fast stretch in dorsiflexion (score: 0, no resistance throughout passive movement; 1, slight resistance throughout passive movement; 2, clear catch at a precise angle, interruption of the passive movement, followed by release; 3, un-sustained clonus occurring at a precise angle; 4, sustained clonus occurring at a precise angle), and the spasticity angle, which measures the R2–R1 difference between the ankle dorsiflexion passive range of motion (R2) and ankle dorsiflexion at which the reaction to fastest stretch (i.e., angle of catch) occurs (R1) [30,31]. The MAS was used to evaluate spastic calf muscles tone. MAS is a 6-point scale grading the resistance of a relaxed limb to rapid passive stretch (score: 0 = no increase in muscle tone; 1 = slight increase in muscle tone at the end of the range of motion; 1+ = slight increase in muscle tone through less than half of the range of motion; 2 = more marked increase in muscle tone through most of the range of motion; 3 = considerable increase in muscle tone; 4 = joint is rigid) [32,33].

### 5.3. DNB Procedures

DNBs were performed with the patient in a prone position, using a 22-gauge, 50 or 80 mm, ultrasound-faceted tip echogenic needle designed for nerve blocks (SonoPlex STIM, Pajunk, Geisingen, Germany). The possible target of the DNB procedure considered in this study were the tibialis nerve motor branches for the GM, GL, soleus, TP, and FDL. Each motor nerve branch was identified using both ultrasound guidance (MyLab 70 XVision system, Esaote SpA, Genoa, Italy; linear probe set at 13 MHz) and electrical nerve stimulation (Plexygon, Vygon, Padua, Italy) [34,35]. Upon the identification of the target nerve, indicated by an appropriate muscle response to electrical stimuli (1 Hz, 100 μs, 0.5 mA), lidocaine 2% was administered. According to the French clinical guidelines for peripheral motor nerve blocks in physical and rehabilitation medicine, the maximum dose of lidocaine administered per DNB session was 2 mg/kg [22]. DNBs were performed one at a time, and their number and sequence were not predetermined but were chosen on a case-by-case basis depending on the clinical assessment. Therefore, the number of DNBs could range from 1 to 5 per affected leg until the expected clinical response was achieved (i.e., Tardieu grade ≤ 2 for both knee extension and flexion).

### 5.4. BoNT-A Treatment

We collected data about BoNT-A dose. To exclude a possible pharmacodynamical interaction with lidocaine, BoNT-A was injected in a separate session than DNB 1–2 weeks later based on the selective DNB procedures (i.e., in the muscles that contributed to clonus according to DNB). BoNT-A injections were guided by ultrasound (MyLab 70 XVision system, Esaote SpA, Genoa, Italy; linear probe set at 13 MHz) and administered using a 22-gauge, 40 mm needle. The dilution ratios were 100 U in 2 mL for Onabotulinum toxin-A and Incobotulinum toxin-A, and 500 U in 2.5 mL for Abobotulinum toxin-A. The dose, number of injection sites, and total dose per session adhered to the label recommendations for each type of BoNT-A.

### 5.5. Data Analysis

Statistical analysis was conducted using the SPSS software package, version 27.0 (SPSS, Chicago, IL, USA). Graphs were created using Microsoft Excel. A descriptive analysis, including mean and standard deviation, was performed for continuous variables (e.g., age, gender, type of illness, time since diagnosis). Frequency analysis, including median and interquartile range (Q1; Q3), was used for categorical variables (e.g., Tardieu grade and MAS). Additionally, percentages and median were used to describe muscle involvement in clonus. The analysis was performed for all patients and separately for each group based on condition (i.e., stroke, MS, and CP). Each leg was considered separately in cases of bilateral involvement (e.g., in MS and CP).

## Figures and Tables

**Table 1:** Demographic and clinical features of enrolled patients.

	Enrolled Patients, (n = 91)
*Demographic features*	
Age, year	
All patients	45.25 ± 18.19
Stroke patients	55.07 ± 13.94
MS patients	50.67 ± 8.81
CP patients	35.28 ± 19.75
Gender, men, n (%)	
All patients	55 (60.4)
Stroke patients	21 (67.7)
MS patients	11 (52.4)
CP patients	23 (59.0)
*Clinical features*	
Etiology of spastic equinovarus foot, n (%)	
Stroke patients	31 (34.1)
MS patients	21 (23.1)
CP patients	39 (42.8)
Spasticity duration, months	
All patients	284.88 ± 253.40
Stroke patients	41.14 ± 48.48
MS patients	92.00 ± 79.90
CP patients	342.46 ± 254.50
MAS knee extended, n	
All patients	3 (2;4)
Stroke patients	4 (3;4)
MS patients	3 (3;4)
CP patients	3 (2;4)
Lower limb right/left, n	67/49 §

**Legend:** Data are expressed as mean ± standard deviation, median (Q1;Q3), or number (percentage) as appropriate. MS: multiple sclerosis; CP: cerebral palsy; MAS: modified Ashworth scale. ^§^ Bilateral involvement is considered separately.

**Table 2:** Tardieu scale score pre- and post-DNB with knee flexed and extended.

	Knee Extended		Knee Flexed	
	Pre-DNB	Post-DNB	Pre-DNB	Post-DNB
Tardieu grade median (IQR)	3 (3;4)	2 (2;2)	3 (3;4)	2 (1;2)

**Legend:** DNB: diagnostic nerve block. Data are expressed as median (Q1;Q3).

**Table 3:** Muscle contributing to clonus according to causes of spasticity.

	Soleus	GM	GL	TP	FDL
All, n (%)	63 (38.2)	28 (17.0)	39 (23.6)	24 (14.5)	10 (6.1)
Stroke, n (%)	19 (43.2)	7 (15.9)	9 (20.4)	8 (18.2)	0 (0)
MS, n (%)	16 (32.7)	11 (22.4)	17 (34.7)	3 (6.1)	2 (4.1)
CP, n (%)	28 (38.9)	10 (13.9)	13 (18.1)	13 (18.1)	8 (11.1)

**Legend:** MS: multiple sclerosis; CP: cerebralpalsy; GM: gastrocnemius medialis; GL: gastrocnemius lateralis; TP: tibialis posterior; FDL: flexor digitorum longus.

**Table 4:** BoNT-A dose and effect on clonus one month after BoNT-A injection.

	Soleus		GM		GL		TP		FDL	
BoNT-A, Unit *	94.3 (19.5)		65.2 (18.2)		73.4 (21.8)		65.2 (18.2)		48.8 (19.5)	
	**Tardieu grade ^§^**					**Score ≤ 2**				
Knee extended	2 (2;3)					55.3%				
Knee flexed	2 (2;3)					50.4%				

**Legend:** BoNT-A: botulinum neurotoxin-A; GM: gastrocnemius medialis; GL: gastrocnemius lateralis; TP: tibialis posterior; FDL: flexor digitorum longus. Data are expressed as mean (standard deviation) or median (Q1;Q3). * Ratio of Onabotulinum toxin-A and Incobotulinum toxin-A to Abobotulinum toxin-A, 1:3 [24]. ^§^ One month after BoNT-A injection.

## Data Availability

The original contributions presented in the study are included in the article, further inquiries can be directed to the corresponding author.
